# The Role of the Lysosomal Cl^−^/H^+^ Antiporter ClC-7 in Osteopetrosis and Neurodegeneration

**DOI:** 10.3390/cells11030366

**Published:** 2022-01-21

**Authors:** Giovanni Zifarelli

**Affiliations:** Institute of Biophysics—CNR, 16149 Genova, Italy; giovanni.zifarelli@ibf.cnr.it

**Keywords:** chloride transport, proton transport, organellar transporter, lysosomal storage disease, osteopetrosis, bone, lysosome, osteoclast

## Abstract

CLC proteins comprise Cl^−^ channels and anion/H^+^ antiporters involved in several fundamental physiological processes. ClC-7 is a lysosomal Cl^−^/H^+^ antiporter that together with its beta subunit Ostm1 has a critical role in the ionic homeostasis of lysosomes and of the osteoclasts’ resorption lacuna, although the specific underlying mechanism has so far remained elusive. Mutations in ClC-7 cause osteopetrosis, but also a form of lysosomal storage disease and neurodegeneration. Interestingly, both loss-of- and gain-of-function mutations of ClC-7 can be pathogenic, but the mechanistic implications of this finding are still unclear. This review will focus on the recent advances in our understanding of the biophysical properties of ClC-7 and of its role in human diseases with a focus on osteopetrosis and neurodegeneration.

## 1. Introduction

**Figure 1:**
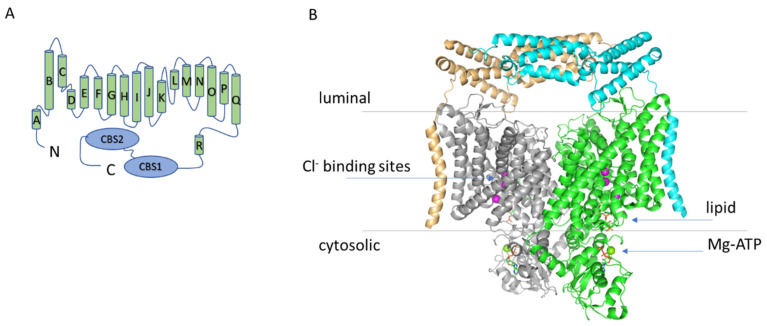
(**A**) Topology diagram of ClC-7. (**B**) Structure of the hClC-7/Ostm1 complex. Structure of the hClC-7/Ostm1 complex based on the work of Schrecker et al. (PDB entry 7JM7) viewed from the membrane plane [6]. The two ClC-7 subunits are represented in grey and green, the two Ostm1 subunits in orange and cyan. The blue arrows indicate the three anion binding sites in the permeation pathway (magenta spheres) and the location of the lipid and of the Mg-ATP (only in the green subunit). The cytosolic C-terminal region of the protein comprises two so called CBS (cystathionine β synthase) domains.

Human CLC proteins comprise Cl^−^ channels and Cl^−^/H^+^ antiporters with fundamental roles in regulating electrical excitability, transepithelial transport and vesicular ionic homeostasis [1,2]. The lysosomal Cl^−^/H^+^ antiporter ClC-7, together with ClC-6, forms a distinct branch of the CLC protein family that is more closely related to the plant homologue AtClC-d than to the other mammalian transporters [3,4]. However, the general structural architecture of ClC-7 is very similar to the ones of the other CLC proteins with an almost identical transmembrane domain and a large cytoplasmic C-terminus comprising two CBS (cystathionine β synthase) domains [5,6] (Figure 1). ClC-7 is ubiquitously expressed with particularly high levels in the central and peripheral nervous system where it colocalizes with Lamp-1, a marker for late endosomes and lysosomes [3,7,8]. Importantly, unlike the other mammalian CLC transporters, ClC-7 requires the β subunit Ostm1 for proper localization and function [9,10,11]. In osteoclasts, ClC-7 is also expressed in the ruffled border, a specialized membrane domain responsible for the acidification of the resorption lacuna, which in turn mediates bone resorption [7]. In lysosomes and in the ruffled border, ClC-7 contributes to the ionic homeostasis, even though the specific role is still debated [9,12,13,14]. Mutations in ClC-7 and Ostm1 cause osteopetrosis [7,9,10], but also a form of lysosomal storage disease and neurodegeneration [8,15,16], consistent with the phenotype of ClC-7 and Ostm1 loss-of-function mouse models [7,10]. ClC-7 expression has also been detected in the luminal membrane of the choroid plexus [17], but its physiological role in that context is still unclear. Intriguingly, *CLCN7* has been identified as the causative gene in a quite unique phenotype combining osteopetrosis, renal tubule acidosis, renal stones, epilepsy, and blindness [18]. Moreover, ClC-7 has been implicated in Alzheimer disease [19]. Interestingly, both loss-of- and gain-of-function mutations of ClC-7 can be pathogenic, although the mechanistic implications of these findings are still unclear. This review will focus on the recent advances in our understanding of the biophysical properties of ClC-7 and of its role in human physiology (in particular in osteopetrosis and neurodegeneration) and will try to provide an integrated perspective from these two fields of investigation.

## 2. The Structure of ClC-7

Two recent cryo-EM studies have elucidated the structure of the ClC-7/Ostm1 complex [5,6]. The association between the two proteins is largely based on interactions of their transmembrane regions at the periphery of the ClC-7 dimer [5,6] (Figure 1). Importantly, the highly glycosylated luminal region of Ostm1 forms a bundle that covers the luminal side of ClC-7, explaining the protective role of Ostm1 against the degradative environment of the lysosomal lumen [5,6]. In other respects, the structure of ClC-7 reproduces most of the key features of the other CLC proteins of known structure [20,21,22,23,24,25]: a dimeric architecture with a transmembrane domain and a large cytosolic region comprising two CBS domains. The ion permeation pathway present in each of the monomers is also very conserved, with a typical “hourglass” shape with a narrowing at the selectivity filter and three anion binding sites (Figure 1).

In particular, in the study of Schrecker et al., a conserved glutamate residue (Glu247 in the hClC-7) with an important role in voltage dependence and Cl^−^/H^+^ coupling in all CLC transporters (so-called “gating glutamate”) is captured with the side chain directed towards the extracellular space and Cl^−^ ions occupying all three binding sites [6] (Figure 1). The structure of the hClC-7 was also investigated by Zhang et al., but they did not identify densities for Cl^−^ ions and could not build an unbiased model for the side chain of the gating glutamate [5]. The pathway for proton movement appears to bifurcate from the Cl^−^ passageway close to the central binding site and be formed by a putatively water-filled cavity around another glutamate residue that is very conserved in mammalian CLC transporters (Glu314 in the hClC-7, so called “proton glutamate”) [5,6], consistent with previous structures and molecular dynamics simulations [26,27,28]. In the cytosolic region, the structure of ClC-7 reveals a previously unrecognized role for the N-terminal domain which interacts both with the transmembrane region and the CBS1 and CBS2 domains forming an extensive intramolecular interaction network [5,6]. Intriguingly, the loop of the N-terminus responsible for this interaction is conserved, among the human CLC transporters, only in ClC-6 [6]. Importantly, the N-terminus in the structures of other CLC proteins was not resolved, probably due to the intrinsic flexibility, and it is, therefore, not possible to conclude whether this role for the N-terminus is unique to ClC-7 or it is relevant also for ClC-6 and other CLC proteins. The CBS domains, with contribution from the N-terminal domain, were found to bind an ATP molecule [6] (Figure 1). The binding coordination was similar to the one observed in the isolated C-terminal portion of ClC-5 [29], but in addition, in ClC-7, a Mg^2+^ ion is also present (Figure 1). Notably, Leisle et al. showed that ClC-7 currents are not affected by ATP [11]. A comparison between the structure of ClC-7 with and without Ostm1 reveals that there are no large-scale differences in the transmembrane region and in ATP and lipid binding (see also below) [6]. However, some subtle structural rearrangements are indeed observed in the permeation pathway and in the structure of the ClC-7/Ostm1 complex the anion occupancy at the central binding site is much lower than in ClC-7 alone [6].

### Phosphatidylinositol Binding Site

Phosphatidylinositol-3-phosphate (PI3P) is a low-abundance constituent of lysosome membranes. Schrecker et al. were able to resolve a molecule of PI3P at the interface between the transmembrane and the cytoplasmic domains [6] (Figure 1). The transmembrane region responsible for the interaction with the phosphate head group is formed by an amphipatic β hairpin between helices αF and αG which is conserved also in ClC-6 and in the plant nitrate/H^+^ antiporter AtClC-a [6], but not in other mammalian CLC proteins. Given the specific lipid composition of lysosomes membranes this finding might be of great biophysical and physiological relevance, but its potential consequences remain to be investigated.

## 3. Cl^−^/H^+^ Exchange and Transport Mechanism in CLC Antiporters

**Figure 2:**
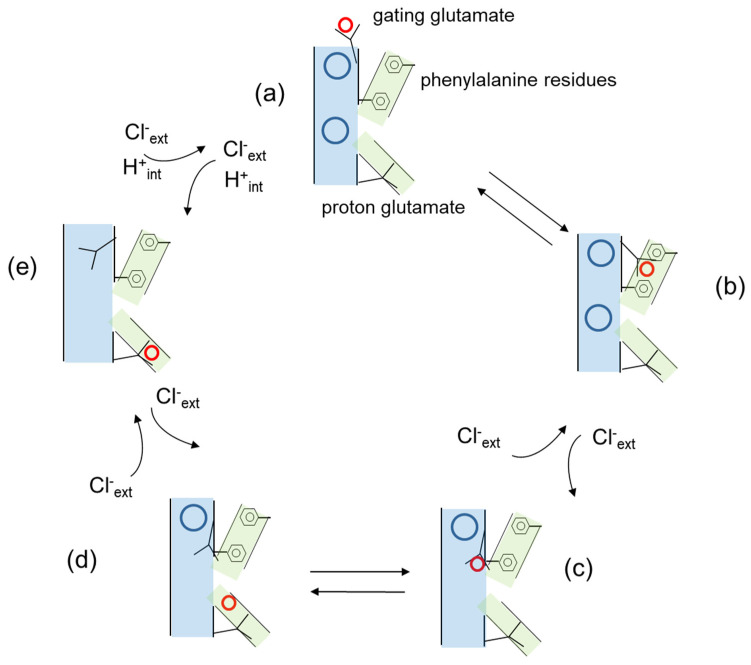
Simplified model of the transport cycle of CLC transporters based on the work of Leisle et al. [27]. Cl^−^ ions at the external and central binding sites are represented as blue circles, H^+^ are represented as red circles, the anion permeation pathway is shown in pale blue, the H^+^ permeation pathway in green. The gating and proton glutamates and the phenyalanine residues are explicitly indicated in the figure. (**a**) The gating glutamate is oriented towards the extracellular space and its side chain protonated. Cl^−^ ions are present at both the external and central binding site. (**b**) The protonated gating glutamate rotates towards the interior of the protein along a pathway defined by two phenylalanine residues. (**c**) The protonated gating glutamate continues its movement towards the central binding site displacing a Cl^−^ ion towards the extracellular space. (**d**) The H^+^ dissociates from the side chain of the gating glutamate. (**e**) The H^+^ binds the proton glutamate and the side chain of the gating glutamate moves upwards to occupy the external anion binding site displacing a second Cl^−^ ion. Not explicitly indicated is an intermediate state following proton release to the cytosolic side in which Cl^−^ ions from the internal side have access to the permeation pathway.

A central aspect of CLC transporter function is the coupled movement of Cl^−^ and H^+^ in opposite directions with a stoichiometry of 2 to 1 [11,12,30,31]. The Cl^−^ and H^+^ pathways are schematically represented in Figure 2, indicating that they are different but intersect at the level of the gating glutamate. Importantly, CLC proteins are unique in that the two transported substrates, Cl^−^ and H^+^, bind simultaneously and not sequentially as in the classical alternate access mechanism common to most transporters [32,33]. At the cytosolic side, Cl^−^ and H^+^ pathways diverge as the proton glutamate that probably serves as a proton acceptor site [34,35] is displaced from the cytosolic opening of the anion permeation pathway [5,6]. Computational studies have also suggested the presence of water-filled protein cavities that might potentially bridge the proton glutamate and the gating glutamate and mediate proton transport [26,36]. At the central binding site, Cl^−^ and H^+^ pathways converge as mutations of the conserved serine residue that coordinates the anion at this site affected both anion selectivity and anion/H^+^ coupling [31,37,38]. Regarding the Cl^−^ and H^+^ pathways from the luminal space to the central binding site, it was assumed for a long time that they would superimpose. Proton would move towards the central binding site as the protonated side chain of the gating glutamate would move from the luminal space to the external and then the central binding site. In this model, proton movement down to the central binding site would result from the competition between the protonated side chain of the gating glutamate and Cl^−^ ions for the external and central binding site [39]. However, very recently Leisle et al. proposed a different model illustrated in a simplified version in Figure 2, in which the movement of the protonated side chain of the gating glutamate does not take place along the Cl^−^ permeation pathway, but rather in the interior of the protein with a critical contribution of two phenylalanine residues, one of which also contributes to the central anion binding site [27]. According to this model, movement of the protonated gating glutamate and the rearrangement of the phenylalanine of the central binding site are coupled to the opening of the intracellular gate formed by conserved serine and tyrosine residues below the central binding site [27].

## 4. Electrophysiological Properties of ClC-7

The electrophysiological investigation of ClC-7 has been possible after the discovery that disruption of N-terminal dileucine lysosomal targeting motifs by alanine substitution produced a partial redistribution of the complex ClC-7/Ostm1 to the plasma membrane [40] and this was sufficient to elicit robust transmembrane current [11,41]. Interestingly, whereas the strong outward rectification of the currents and the inhibitory effect of acidic extracellular pH are properties that ClC-7 shares with other CLC transporters [11,41], there are also important differences. In fact, ClC-7 displays a very slow activation kinetics with a time constant in the order of seconds whereas for the other CLC transporters investigated so far the activation is to a large extent instantaneous [11,41]. However, it should be kept in mind that these are properties observed upon expression of ClC-7 at the plasma membrane, and these might differ from the ones in the physiological location, the lysosomal membrane, in particular, because of the different lipid composition and the observed PI3P binding to ClC-7 [6], but also due to the potential presence of unidentified lysosomal binding proteins.

### 4.1. The Role of the Proton Glutamate

Another important difference is related to the function of the so-called “proton glutamate”, E312 in rClC-7. Neutralization of this conserved residue in ClC-3, ClC-4, ClC-5 and ClC-6 ablates transport activity [42,43,44]. Under the assumption that in ClC-7 neutralization of the proton glutamate would have the same effect, Weinert et al. generated a knock-in mouse model (so called transport deficient, or “td”) carrying the E312A mutation to explore possible roles of ClC-7 that were unrelated to ion-transport [45]. Indeed, the mutation resulted in an osteopetrosis that was as severe as in the ClC-7 KO mice but with milder neurodegeneration and no defect in pigmentation, suggesting that there was also a functional role for a transport-deficient ClC-7, for example, in contributing to the assembly of a lysosomal macromolecular complex [45]. Leisle et al. later suggested that indeed the E312A mutation ablates transport current in ClC-7 [11]. However, a detailed electrophysiological characterization recently showed that this mutant does mediate transport currents, although their magnitude is strongly reduced compared to WT [41]. This finding potentially explains the phenotype of the E312A knock-in mouse model, as the residual current mediated by the E312A mutant could rescue the pigmentation phenotype and ameliorate the neurodegeneration compared to a full ClC-7 KO. However, further studies are required to fully clarify this point.

### 4.2. Transient Capacitive Currents

The work of Pusch et al. also suggested another specific feature of ClC-7 related to the mechanism that originates the transient capacitive currents [41]. Proton glutamate neutralizing mutations in ClC-3, ClC-4 and ClC-5 not only ablate transport current, but are also associated with either the appearance or an increase in magnitude of transient capacitive currents [44,46,47,48,49]. A detailed analysis of transport current in ClC-5 suggested that the protein switches between transport-incompetent (or “inactive) and transport-competent (or “active”) states [42,48], leading to the proposal that the transient capacitive currents observed in the proton glutamate mutations would reflect charge movement associated with gating transitions (transition between inactive and active state) rather than transport activity. For ClC-7, the situation appears very different. First of all, the activation kinetics of transport currents in ClC-7 is order of magnitude slower than in ClC-5 (seconds vs. milliseconds) [11,41]. Moreover, in ClC-7, the transient current appears to be linked to the transport cycle itself rather than to a gating process that precedes transport since the transient current amplitude is independent of the fraction of activated transporters and it is the same in the WT and in the mutant R760Q although the activation kinetics of the mutant is much faster [11,41].

## 5. Osteopetrosis

**Figure 3:**
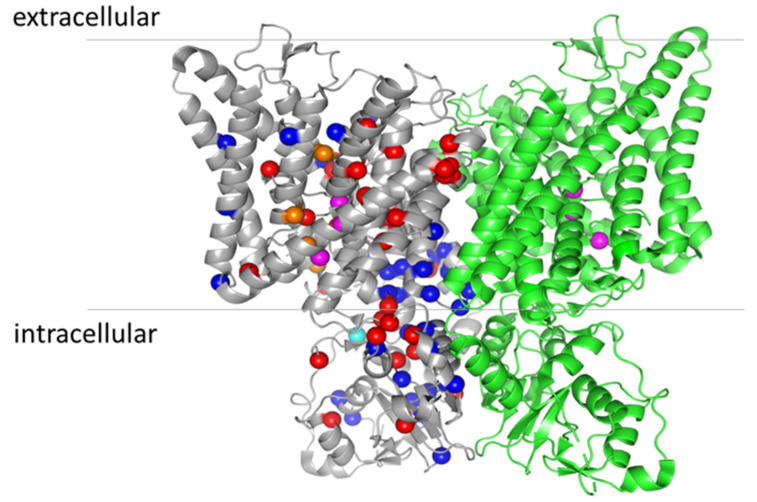
Location of osteopetrosis mutations on the structure of ClC-7 (PDB entry: 7JM7). For clarity, the mutations are indicated only in the grey monomer. ARO mutations are represented by red spheres, ADO II mutations by blue spheres and IARO mutations by orange spheres (except the variants found as compound heterozygous V418M-R674Q, as they also cause ADO II when expressed alone [60]). The cyan sphere represents the mutation Y715C, causing lysosomal storage disease and albinism without osteopetrosis [68]. The magenta spheres represent the three anion binding sites in the permeation pathway.

Physiological bone tissue remodeling requires a balance between bone formation and resorption mediated by osteoblasts and osteoclasts, respectively. In osteopetrosis this balance is disturbed as bone resorption is impaired resulting in dense but fragile bones [50]. The molecular mechanism of bone resorption by osteoclasts is based on a specialized domain, the ruffled border, formed in the area where the osteoclasts tightly seal on the bone matrix. Protons and secretory lysosomes containing bone-resorbing enzymes are released into the ruffled border to attack both the inorganic and organic matrix of bones [51]. In particular, osteoclast-rich osteopetrosis indicates a form of osteopetrosis in which the number of osteoclasts is not reduced, and osteoclasts actually have a higher survival rate and increased surface area, most probably due to the reduced release of proapoptotic signals during bone resorption [51]. Osteoclast-poor osteopetrosis indicates an osteopetrosis caused by a reduced number of osteoclasts and with a milder phenotype compared to the osteoclast-rich form. Classically, two main forms of osteoclast-rich osteopetrosis can be distinguished, depending on the inheritance pattern: autosomal dominant osteopetrosis (ADO, or OPTA) and autosomal recessive (ARO, or OPTB). However, they are both heterogeneous from the genetic, mechanistic, and phenotypic point of view, and correspondingly they comprise a broad spectrum of clinical manifestations [52,53]. Here, we will discuss in more detail the forms of osteopetrosis caused by mutations in ClC-7 (Figure 3 and Table 1).

### 5.1. Autosomal Dominant Osteopetrosis Type II (ADO II)

Regarding the types of osteopetrosis due to ClC-7 dysfunction, ADO type II (also OPTA2 or Albers-Schonberg disease type II) is the most common form with a prevalence of 0.2 to 5.5 in 100.000 [54]. It is usually diagnosed in late childhood/adolescence, and typical symptoms include non-traumatic fractures and skeletal-related events such as nerve compression syndrome (leading to partial visual loss) and bone marrow failure, but only rarely the is disease life-threatening [55,56]. In radiographies, it appears as segmentary osteosclerosis, predominantly at the vertebral endplates (‘rugger-jersey spine’), iliac wings (‘bone within bone’ sign), and skull base [54,56]. The disease is associated in 70% of the patients with heterozygous missense mutations in ClC-7, whereas for the remaining 30% there is no clear genetic association [57]. While ADO II is considered largely a bone disease, both old and new evidence indicates very important extra-skeletal manifestations, a fact which is actually consistent with the broad ClC-7 tissue distribution [3,15,58]. One indication of such extra-skeletal manifestations is the long-known increase in creatine kinase observed in several osteopetrosis patients, which suggests a myopathy phenotype [59]. Interestingly, a detailed investigation of heterozygous G213R mice, representing a validated model of human ADO II, revealed that they also have several extra-skeletal manifestations, showing anxiety, depression, β-amyloid accumulation, and astrogliosis, suggesting a relevant involvement of the nervous system [57]. In addition, lung, kidney, spleen and muscle are also affected through macrophage infiltration and activation of fibrotic signaling [57]. Confirming these conclusions, an siRNA approach against the mutated ClC-7 was effective in improving the extra-skeletal phenotypes [57]. It is intriguing to correlate this conclusion with the findings of Rossler et al. about an ARO patient, compound heterozygous for the mutations G292E and R403Q, who presented brain abnormalities disproportionally severe in comparison to the osteosclerosis [60] (see also below). This patient died at the age of 14 months of respiratory failure. Analysis of patient hiPSCs from blood cells showed several interesting findings. In particular, an increase in the autophagy marker LC3-II in undifferentiated hiPSCs, which was also present in tissue-specific *Clcn7* KO mice [15]. Defective autophagy is indeed emerging as an underlying mechanism in several neurodevelopmental disorders [61,62], and it was also observed in the mouse model carrying the heterozygous *Clcn7*^G213R^ mutation (homologue of the human, G215 mutation causing ADOII) [57]. Moreover, the differentiated patient osteoclasts had a larger diameter and a higher number of nuclei compared to osteoclasts differentiated from controls, a finding already observed in both Ostm1 and ClC-7 deficient mice but still not fully understood [63,64]. The differentiated osteoclasts also showed a complete loss of bone resorption activity and upon heterologous expression and electrophysiological measurements the mutations showed an abolished (G292E) and strongly reduced (R403Q) ionic current, consistent with a fundamental loss of function as the basis of the disease [60].

### 5.2. Autosomal Recessive Osteopetrosis (ARO)

ARO (or OPTB4) has a prevalence of 1:250,000 in the general population, but it is much more frequent in some ethnic groups (for example in Costa Rica and northern Sweden) [65]. ARO patients have a generalized increase in bone density leading to macrocephaly, growth retardation, eye protrusion (exophthalmos), small jaw (micrognathia) and hypertelorism (increased distance between the eyes). The most severe forms of ARO are often neuropathic due to primary neurodegeneration with symptoms ranging from developmental delay to hypotonia, retinal atrophy and seizures [50,66]. Other symptoms are due to the constriction of bone marrow space like anemia, thrombocytopaenia, compensatory extramedullary hematopoiesis, hepatosplenomegaly, and recurrent infections [67]. ARO is also often associated with low serum Ca^2+^ and secondary hyperparathyroidism [68]. It is usually diagnosed at birth or early infancy, and it is often lethal in early life if not treated with hematopoietic stem cell transplantation (HSCT), but this holds only if the therapy is initiated before CNS involvement [68,69]. It is estimated that ClC-7 is responsible for 10–15% of ARO cases [52].

## 6. The Physiological Role of ClC-7 and Ostm1

The first indication of a role of ClC-7 in osteopetrosis came from the phenotype of ClC-7 knockout mice which recapitulated the human disease: *Clcn7*^−/−^ mice showed severe osteopetrosis and retinal degeneration [7]. In particular, KO mice were smaller, had dysmorphic heads, abnormal body posture and short limbs. Excessive bone density was particularly evident in long bones which lacked a bone marrow cavity and in the failure of teeth to erupt. The survival was also affected and limited to 6–7 weeks.

As commonly observed in patients, the decrease in bone marrow caused hepatosplenomegaly secondary to extramedullary blood production also in KO mice. Retinal degeneration is often associated with osteopetrosis and was recapitulated also in KO mice. Further analysis revealed that osteoclasts were present, but developed only rudimentary ruffled borders and were unable to resorb bone in vitro because of the inability to acidify extracellular compartments [7]. The causative role of ClC-7 in human osteopetrosis was confirmed in the same study by the identification of a patient affected by ARO who was compound heterozygous for the nonsense mutations Q555x and the R762Q substitution in ClC-7 [7]. A similar mechanism is the basis for the ARO phenotype showed by the grey-lethal mouse line, harboring an inactivating mutation of Ostm1 [10]. Besides osteopetrosis, ClC-7 and Ostm1 deficient mice also have a pigmentation phenotype, which also indicates an additional physiological role [7,10]. As mentioned above, mutations in ClC-7 and Ostm1 can cause both ADO or ARO [7,10], both characterized by a spectrum of phenotypic presentation that makes the classification quite difficult when no detailed genetic data are available for the affected families [52]. In fact, there are patients that escape this schematic classification, and several ADO II mutations are present in families in which some of the carriers remain asymptomatic for the entire life whereas others develop osteopetrosis of varying degrees of severity [52,54,70,71,72,73,74]. The intermediate recessive form of the disease (IARO) described by some authors does not have a clear-cut classification, and even the mode of inheritance is questioned [52,54,74,75]. So far, only five patients have been identified with this subtype, three with the homozygous mutations G203D and P470Q [76], and P470L [77], and the two compound heterozygous L224R-K691fs [71] and V418M-R674Q [70]. In these patients, IARO is characterized by a relatively mild generalized increase in bone density with spontaneous fractures in the first years of life, mandibular prognathism, osteomyelitis, anemia, hepatosplenomegaly, and occasional optical nerve compression, but have a longer life expectancy compared to ARO [71,76]. ARO patients with mutations in either ClC-7 or Ostm1 might develop the neuropathic subtype of the disease associated with a particularly poor prognosis. Interestingly, the G215R heterozygous mutation in ClC-7 has been found in a family showing ADO II of variable expressivity and an unusual syndrome comprising renal tubular acidosis, renal stones, developmental delay, blindness, and epilepsy [18]. Intriguingly, the G215R mutation has been previously identified in several families with classical ADO II, supporting the notion of poor genotype–phenotype correlation for some ClC-7 mutations causing osteopetrosis [52,54,74].

### ClC-7 Molecular Role from Animal and Cellular Models

**Figure 4:**
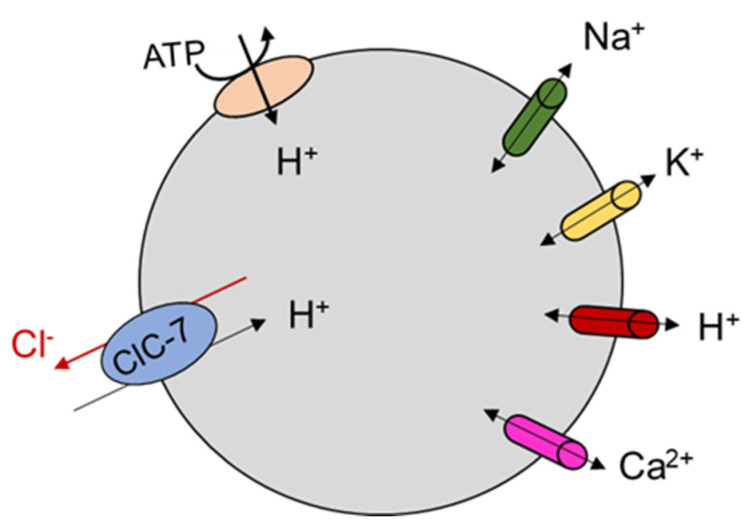
Schematic representation of the role of ClC-7 in lysosomes. Lysosomal acidification results from the complex interplay of anion and cation transport mediated by different families of ion channels and transporters. ClC-7 is indicated in light blue, V-type ATPase in light red. A variety of cation channels is also indicated. The orientation of Cl^−^ and H^+^ movement is suggested on the basis of the electrophysiological studies in which ClC-7 is expressed at the plasma membrane.

It has been speculated for a long time that vesicular CLC proteins were chloride ion channels needed to balance positive charge accumulation produced by active proton transport into the organellar lumen, allowing for effective acidification of these compartments (reviewed in [14]). The discovery that ClC-7 is a Cl^−^/H^+^ antiporter suggested a more complex physiological role which is still debated [11,12] (Figure 4). Osteoclasts of ClC-7 KO mice failed to acidify the resorption lacuna [7] even though lysosomal pH in neurons and other cell types in ClC-7 and Ostm1 deficient mice was normal [8]. A mouse line homozygous for the uncoupling mutation E245A, so-called ClC-7^unc/unc^, which turns ClC-7 into a pure Cl^−^ conductance (i.e., transforms ClC-7 from a Cl^−^/H^+^ antiporter to a Cl^−^ channel), made it possible to investigate the specific role of Cl^−^ transport in the regulation of lysosomal pH [13]. In comparison to ClC-7 KO mice, these mice presented a milder osteopetrosis but a similar lysosomal storage disease and no change in fur color [13].

In particular, lysosomal pH was normal, as in ClC-7 KO, but luminal [Cl^−^] was reduced. This suggests a specific physiological role for Cl^−^/H^+^ exchange activity in lysosomes. In contrast to this, a role for ClC-7 as shunt conductance enabling lysosomal acidification by the V-type ATPase is suggested by the impaired lysosomal acidification in cells in which ClC-7 was knocked down by siRNA [12], and by the observation that the Y715C gain of function mutation led to lysosomal hyper-acidification [78]. As explained in paragraph 4.1, in the E312A homozygous knock in mouse line (which probably has a reduced level of transport current) osteopetrosis is as severe as in ClC-7 KO mice, but neurodegeneration is milder and there is no pigmentation phenotype [45]. As for ClC-7 KO and for the ClC-7^unc/unc^, also the E312A homozygous mice had lysosomes with normal pH but reduced luminal [Cl^−^] [45]. These findings highlight the physiological importance of the lysosomal [Cl^−^], but also underscore the fact that general ion homeostasis (including also protons, potassium, calcium and sodium ions) in this compartment is determined by the complex interplay between ClC-7-mediated transport and several types of cation ion channels that have been newly identified at an ever-increasing pace in the last decade [79,80] (Figure 4). For example, the voltage across the lysosomal lumen is contributed by all the types of ion channels and transporters expressed in lysosomes, but in turn will also influence their activity, particularly in the case of voltage-dependent proteins [81,82]. A critical role for cation conductances in determining lysosomal pH had originally been suggested on the basis of anion substitution experiment in the seminal work of Steinberg et al. [83], and in two early pioneering works [84,85]. An increasing level of complexity with the interplay of multiple ion channels and transporters has also been suggested in a model of osteoclast resorption lacuna acidification [86].

Two mouse models of ADOII have been generated with the human mutations G215R (G213R in mouse) [87] and F316L [88]. Considering the phenotypic variability and the incomplete penetrance of human ADOII, estimated to be around 66% [89] it is interesting to note that the work of Alam et al. suggested an effect of the genetic background on the severity of the osteopetrosis symptoms of the G213R heterozygous mice [87] further supporting a role of genetic modifiers in determining the severity of the disease.

Besides KO and KI mouse lines, human induced pluripotent stem cells (hiPSCs) are also a valuable model for human diseases, and important developments have been achieved in the optimization of the differentiation protocol and functional characterization of ARO patient-derived osteoclasts [60].

## 7. Lysosomal Storage Disease and Neurodegeneration

Severe ARO cases are often associated with neurodegeneration manifesting in developmental delay, hypotonia, retinal atrophy and seizures [66,90,91] consistent with the finding that ClC-7 KO mice displayed neurodegeneration, with the highest impact in the hippocampus, the cortex, and the cerebellum [8]. In addition, these KO mice displayed hallmarks of neuronal ceroid lipofuscinoses (NCL) a form of lysosomal storage disease. However, the lysosomal pH of ClC-7 KO cultured neurons did not differ from WT. Neurodegeneration in ClC-7 KO mice was accompanied by microglia activation and astrogliosis, a common finding in CNS pathologies. This was indicated by overexpression of genes involved in the immune response of microglia [8] similar to what was found in mouse models of mucopolysaccharidoses, a lysosomal storage disease associated with neurodegeneration [92], and in the G213R mouse model of ADO II [57]. Like in these diseases, microglia initial activation against neuronal pathology might lead to a paradoxical response with adverse effects [93]. The retinal degeneration observed in ClC-7 KO mice is probably also due to lysosomal dysfunction [8], another finding common in NCLs. Importantly, the neurological defects were not present in a different mice model for osteopetrosis with mutation in the a3 subunit of the V-type H^+^ pump [94,95]. Conditional ClC-7 KO mice and tissue-specific analysis directly revealed that accumulation of lysosomal storage material is intrinsic to cells lacking ClC-7, and that the massive activation of microglia and astrocytes is limited to brain regions where ClC-7 was deleted [96]. In the same study, it was shown that in these mice the lack of ClC-7 in proximal tubule cells did not affect the endocytic activity but drastically reduced (but did not abolish) proteolysis of endocytosed protein [96].

Grey-lethal (*gl*) mice also showed hallmarks of lysosomal dysfunction, with accumulation of sphingolipids in the brain and increase in the autophagic marker LC3-II observed also in ClC-7 KO [96,97]. The role of ClC-7 and other CLC transporters in neurodegeneration has recently been discussed in detail by Bose et al. [16]. Interestingly, the de novo ClC-7 Y715C heterozygous mutation described in two unrelated children did not cause ADO, but a pleiotropic syndrome including albinism, developmental delay, organomegaly, and lysosomal storage [78]. Functional analysis of this mutation showed a gain-of-function with much larger currents upon expression in Xenopus oocytes and a more acidic pH of the lysosomes of patient-derived fibroblasts, which were also characterized by enlarged cytoplasmic vacuoles [78]. It is instructive to compare these findings with some recent discoveries highlighting the role of ClC-6, a late endosome CLC transporters closely related to ClC-7 and with partially overlapping localization [98,99]. ClC-6 deficient mice lack an obvious phenotype beside a very mild late onset neurodegeneration [91,98]. However, the heterozygous Y533C mutation was recently found in three patients (all heterozygous) affected by variable early-onset neurodegeneration with brainstem lesions and cortical or cerebral atrophy, respectively [100]. This mutation results in a gain of function in terms of transport current producing, when expressed in heterologous systems, a vacuolation phenotype that is similar to the one observed in the Y715C in ClC-7. However, a critical difference between the two mutations is that they lead to opposite effects on the acidification of the enlarged organelle which is increased in patient derived lysosomes with the Y715C mutation in ClC-7 but decreased in lysosomes of cells transfected with the Y533C mutation in ClC-6 [78,100]. Very recently, the ClC-6 mutation E200A was identified in patients with early infantile epileptic encephalopathy West syndrome, and it has been found to impair autophagy [101] similar to the effect of ClC-7 KO [96].

## 8. Structure–Function Analysis of ClC-7

Several functional analyses of disease causing mutations have been conducted [7,11,78,102,103], and in combination with the very recent structures of the ClC-7/Ostm1 complex [5,6], this provides the opportunity to test if there is any correlation between alterations of ClC-7 functional properties and the phenotype of the disease. The first observation that emerges from the mapping of ARO and ADO II mutations onto the structure of ClC-7 is that there is no single hot-spot, and mutations are distributed throughout the whole protein. A general functional feature is that the mutations that cause osteopetrosis often affect the activation kinetics, most probably through an effect on the common gate. The term common gate refers to the mechanism that controls the level of transport activity of ClC-7 in a voltage- and pH-dependent manner [14,104]. The attribute common describes the fact that it simultaneously controls the activity of both monomers of the dimeric ClC-7 (reviewed in [1]). The molecular underpinning of the common gate is still unresolved but it likely involves large conformational changes [105]. In particular, for ClC-7, it has been shown that the common gate depends on the subunit interface and both the transmembrane region and the cytoplasmic domains in the two monomers [104]. The wide distribution of osteopetrosis mutations with effects on the common gate is consistent with the notion of a large conformational change. In particular, the dimer interface between the CBS2 domains is contributed by the polar amino acids S753, R756, Lys759 and Asn774 from one monomer and Ser744, Tyr746 and Asn776 from the other monomer [5], and they are either directly involved in osteopetrosis, like Ser744, Tyr746 and S753, or are very close to residues that when mutated cause osteopetrosis (see Table 1).

Additional residues involved in osteopetrosis and located at the dimer interface are in the CBS1 domains like R674Q, G677V. Other residues in the transmembrane regions close to the dimer interface are R126, P376. M332 and P582 (ARO) and W127 F318, W319 R326 and G347 (ADO II) (Figure 2). It is important to underscore that there are also some ARO, and ADO II mutations located at the periphery of the dimers, or in any case far from the dimer interface, like I261 and R403 (ARO) and R409, V418 and S473 (ADOII). Moreover, ARO and ADO II mutations do not segregate, and in a few cases, mutation of the same residue causes ARO or ADO II depending on the specific amino acid substitution (see Table 1 and Figure 2). A specific structural feature of ClC-7 is that unlike the structures of other CLC proteins, the N-terminus is resolved and contributes to the subunit interface forming a loop with polar interactions with the transmembrane domain and the CBS2 domain [5]. The residue Y99 in this region is mutated to C in patients with ADO II [74], and several mutations of this and nearby residues produce a marked acceleration of the gating kinetics [5]. Moreover, several osteopetrosis mutations involve residues that form polar interactions between N-terminus, transmembrane domain and CBS2, like R286Q, R762Q, R762L and R767Q [5,6]. From these findings, Zhang et al. concluded that the common gate in ClC-7 relies on an extensive interaction among different protein region, including the dimer interface, and that this increases the kinetic barrier for the voltage activation of the common gate. In such a model, osteopetrosis mutations affecting this interaction and decreasing the energy barrier would result in faster kinetics [5]. In relation to the genotype–phenotype correlation of ARO, Di Zanni et al. observed that some ARO mutations associated with neurodegeneration (R126H, A299V/E, P582H and G780R) reduced lysosomal localization and produced no or little current when expressed at the plasma membrane. In contrast to this, ARO mutations without neurodegeneration (L90P, P376L, A511T, G780W/R, A590T and R791C) preserved ion transport activity [102]. While these are interesting observations, it should be kept in mind that the protein localization is often altered by overexpression in heterologous systems. Moreover, the mechanism of dominance in ADO II is not yet firmly established. It might derive from a dominant negative effect of the mutated subunit on the kinetics of the dimeric protein (gain-of-function) similar to the mechanism by which heterozygous mutations in the muscle channel ClC-1 cause dominant (Thomsen) myotonia [120]. However, the dominant effect could be exerted also through a loss-of-function, by hampering the correct localization of the dimer or its stability. For example, while the mutations R762Q [11] and Y746Q [103] both cause an accelerated kinetics, for R762Q, the protein was unstable in patient-derived fibroblasts [7], whereas Y746Q has normal expression level and lysosomal localization [103]. Another layer of complications might be conferred by the discovery that ClC-7 binds ATP and lipids [6]. In this respect, it is interesting to note that several mutations leading to osteopetrosis (Gly765, Leu766, Arg767) map on CBS2 near the ATP-binding site [6] (Table 1 and Figure 2). It is possible to speculate that the different phenotypes of R767 mutations, with R767P and R767W displaying almost no functional activity and R767Q displaying faster activation kinetics might be due to a different impact of the mutations on gating and/or ATP binding. Mutation of Tyr715, located near the PI3P binding site, causes a novel lipid storage disease without osteopetrosis [78]. At the functional level the mutation produces a gain-of-function with increased current level and hyper-acidification of the lysosomes, but no major changes in current kinetics. In conclusion, the mechanism explaining why both gain-of-function and loss-of-function mutations of ClC-7 cause osteopetrosis and why mutations affecting the common gate cause both ARO and ADO remains to be elucidated. The great progress achieved in the electrophysiological investigation of ClC-7 when expressed at the plasma membrane will be ideally combined in the future with techniques that allow to study its properties in its physiological location, the lysosomal membrane, to better understand its contribution to the organellar ionic homeostasis.

## Figures and Tables

**Table 1:** List of disease-causing CLCN7 mutations.

Mutation	Amino Acid Location	Disease	Current Amplitude	Current Activation	Lysosomal Localization	
L90P (splice variant)	N-terminus	ARO	normal	normal	normal	[102]
Y99C	N-terminus	ADO II				[74]
R126H	Helix B	ARO (neurodegen.)	reduced	accelerated	strongly reduced	[102]
W127G	Helix B	ADO II				[106]
L132P (L227del)	Helix B	ARO				[72]
D145fs	Helix B	ARO				[52]
D145G	Helix B	ADO II		accelerated	normal	[107]
W179x	Helix C	ADO II				[52]
G203D	Loop helix C-D	IARO				[76]
L213F	Helix D	ADO II	normal	accelerated		[11,108]
N214S (R767P)	Helix D	ARO				[72]
G215R	Helix D	ADO II and ADO II + renal tubular acidosis			ER retention	[18,52,54,109,110]
L224R (K691fs)	Helix E	IARO				[71]
G240E (W127G)	Helix E	ARO				[111]
G240R (A299E)	Helix E	ARO (neurodegen.)	strongly reduced		reduced	[11,102]
G240R (R526W)	Helix E	ARO				[52]
G240R (L651P)	Helix E	ARO				[72]
P249L	Helix F	ADO II				[54,102]
P249R (S744F)	Helix F	ARO				[52]
I261F	Helix F	ARO				[112]
R271x	Loop helix F-G	ARO				[72]
R280C (splice variant)	Loop helix F-G	ARO				[113]
R286Q	Helix G	ADO II	normal	accelerated		[11,52]
R286W	Helix G	ADO II				[71,108]
V289L	Helix G	ADO II				[114]
S290F	Helix G	ADO II				[106]
S290Y	Helix G	ADO II				[71]
G292E (R403Q)	Helix G	ARO (neurodeg.)				[60]
V297M	Helix G	ARO	strongly reduced		normal (increased overall expression)	[52,115]
A299E	Helix G	ARO (neurodegen.)	strongly reduced		strongly reduced	[102,116]
A299V	Helix G	ADO II/ARO (neurodegen.)	strongly reduced		strongly reduced	[102]
E313K	Helix H	ADO II				[106]
A316G	Loop helix H-I	ADO II				[106]
F318L	Loop helix H-I	ADO II	reduced		normal	[52,72]
F318S	Loop helix H-I	ADO II				[111]
W319R	Loop helix H-I	ADO II				[71]
L323P	Helix I	ADO II	normal	accelerated	normal	[102]
R326G	Helix I	ADO II				[71]
M332V (R767W)	Helix I	ARO				[52]
G347R	Helix I	ADO II				[71]
E374x (in frame insertion G306)	Loop helix I-J	ARO				[52]
P376L	Helix J	ARO	reduced	accelerated	strongly reduced	[102]
R403Q (G512R)	Helix J	ARO				[72]
R409W	Loop helix J-K	ADO II				[117]
V418M	Helix K	ADO II				[70]
V418M (R674Q)	Helix K	IARO				[70]
V418fs	Helix K	ARO				[72]
P470L	Loop helix K-L	IARO				[77]
P470Q	Loop helix K-L	IARO				[76]
S473N	Helix L	ADO II				[71]
L490F	Helix M	ADO	reduced		Normal (reduced overall expression)	[11,52]
C502Y (V577M)	Helix M	IARO				[118]
A511T (G780W)	Loop helix M-N	ARO				[102]
G521R	Helix N	ARO (neurodegen.)	strongly reduced		reduced	[52]
R526Q	Helix N	ARO				[72]
R526T	Helix N	ARO				[72]
R526W	Helix N	ARO	strongly reduced		reduced ER retention	[52]
L549P	Helix O	ARO				[72]
Q555x (R762Q)	Helix O	ARO				[7]
R561Q	Loop Helix O-P	ARO				[119]
L564P	Helix P	ADO II				[71]
P582H	Helix Q	ARO (neurodegen.)			reduced	[102]
A590T	Helix Q	ARO			normal	[102]
L614P (Del exon 17)	Loop helix R–CBS1	ARO				[52]
P619L	Loop helix R–CBS1	ARO	reduced			[52,115]
P634fs	CBS1	ARO				[72]
L651P	CBS1	ARO	strongly reduced		normal	[52]
R674Q	CBS1	ADO II				[70]
G677V	CBS1	ADO II				[52,54]
K689E	Loop CBS1-CBS2	ADO II				[54]
K691E	Loop CBS1-CBS2	ADO II	reduced	slower	reduced	[102]
R712x (E730x)	Loop CBS1-CBS2	ARO				[72]
Y715C	Loop CBS1-CBS2	Lysosomal storage + albinism				[78]
G741R	Loop CBS1-CBS2	ADO II				[106]
R743W	Loop CBS1-CBS2	ADO II				[106]
S744F	Loop CBS1-CBS2	ARO	normal			[11,52]
Y746Q	CBS2	ADO bovine		accelerated	normal	[103]
S753W	CBS2	ADO II				[111]
F758L	CBS2	ADO II				[72]
R762L	CBS2	ADO II		accelerated		[11,108]
R762Q	CBS2	ARO		accelerated		[7,11]
R762W (splicing variant)	CBS2	ADO II				[72]
L766P	CBS2	ARO				[54]
R767P	CBS2	ARO	strongly reduced		normal (reduced overall expression)	[11]
R767Q	CBS2	ARO	normal	accelerated		[11,52]
R767W	CBS2	ADO II	Strongly reduced		Normal (reduced overall expression)	[11,54]
R767W (M332V)	CBS2	ARO				[52]
G780R (splice variant)	CBS2	ARO (neurodegen.)				[102]
A788D	CBS2	ADO II	normal			[11,73]
R791C	CBS2	ARO	normal	accelerated	strongly reduced	[102]
G796fs	CBS2	ADO II		accelerated		[11,54]

The table indicates the amino acid location, the specific type of osteopetrosis (ARO, ADO II and IARO), the effect of the mutation on the current amplitude and activation kinetics and on the lysosomal localization.
